# Oncological outcomes of laparoscopic versus open gastrectomy after neoadjuvant chemotherapy for locally advanced gastric cancer: a retrospective multicenter study

**DOI:** 10.1186/s12957-021-02322-2

**Published:** 2021-07-09

**Authors:** Islam Khaled, Pablo Priego, Hany Soliman, Mohammed Faisal, Ihab Saad Ahmed

**Affiliations:** 1 Surgical Oncology Unit, Department of Surgery, Faculty of Medicine, Suez Canal University Hospital, Kilo 4.5, Ring Road, Ismailia, Egypt; 2 Department of Oesophagogastric, Bariatric and Minimally Invasive Surgery, Ramon y Cajal University Hospital, Madrid, Spain; 3 Department of Clinical Oncology, Faculty of Medicine, Cairo University, Cairo, Egypt; 4 Surgical Oncology Department, National Cancer Institute, Cairo University, Cairo, Egypt

**Keywords:** Gastric cancer, Chemotherapy, Laparoscopy, Gastrectomy

## Abstract

**Background:**

The oncological outcomes of laparoscopic gastrectomy (LG) and open gastrectomy (OG) following neoadjuvant chemotherapy have been investigated in a few studies. Our purpose was to evaluate the oncological outcomes of LG and OG after neoadjuvant chemotherapy in patients with locally advanced gastric cancer (GC) and to determine the advantages, preferences, and ease of use of the two techniques after chemotherapy.

**Methods:**

We conducted a retrospective chart review of all patients who underwent either OG (*n* = 43) or LG (*n* = 41). The neoadjuvant treatment regimen consisted of capecitabine plus oxaliplatin for three cycles, which was then repeated 6 to 12 weeks after the operation for four cycles.

**Results:**

The hospital stay time and intraoperative blood loss in the LG group were significantly lower than those in the OG group. The mortality rate and the 3-year survival rate for patients in the LG group were comparable to those of patients in the OG group (4.6% vs. 9.7% and 68.3% vs. 58.1%, respectively). Similar trends were observed regarding the 3-year recurrence rate and metastasis. The mean survival time was 52.9 months (95% confidence interval [CI], 44.2–61.6) in the OG group compared with 43.3 (95% CI, 36.6–49.8) in the LG group. Likewise, the mean disease-free survival was 56.1 months (95% CI, 46.36–65.8) in the LG group compared with 50.9 months (95% CI, 44.6–57.2) in the OG group.

**Conclusion:**

LG is a feasible and safe alternative to OG for patients with locally advanced GC receiving neoadjuvant chemotherapy.

**Supplementary Information:**

The online version contains supplementary material available at 10.1186/s12957-021-02322-2.

## Introduction

Gastric cancer (GC), which affects > 950,000 patients annually, is the fifth most prevalent cancer and the third most common cause of cancer-related death worldwide [1–3]. Epidemiological studies have shown that the overall incidence of GC is decreasing, likely because of changes in lifestyle, such as lower salted and preserved food intake and reduced *Helicobacter pylori* infection. Advanced GC is identified when the tumor invades beyond the submucosal layer, even without metastasis and N0 staging [4]. The 5-year survival after GC diagnosis ranges from 70% for stage Ia to 5% for stage IV [5]. In addition, the choice of the treatment strategy, such as potentially curative treatment, endoscopic treatment, or palliative treatment, depends on the disease stage.

Laparoscopic gastrectomy (LG) is one of the standard procedures for early GC and has also proven its feasibility in locally advanced GC [6–8]. Because of its low invasiveness, shorter hospitalization duration, faster bowel movement recovery, and good cosmetic outcomes, LG has recently gained great popularity for the management of early GC [9–13]. Many systematic reviews have proven the feasibility of LG compared with open gastrectomy (OG) in patients with GC [12, 14–17]. Intraoperative circulatory and respiratory disturbances and the longer operative time are the main issues in LG-related difficulties; in addition, the tissue-related factors after neoadjuvant chemotherapy lead to avoidance of laparoscopic gastrectomy following neoadjuvant chemotherapy [18–20]. The majority of randomized clinical trials (RCTs) that compared LG and OG for early GC have reported early findings on the procedural safety of LG and its short-term benefits [21–23]. In terms of advanced GC, there is insufficient evidence from comparisons of LG and OG, particularly in patients receiving neoadjuvant chemotherapy.

On the other hand, a multimodality approach is the cornerstone for management of patients with advanced GC. Currently, adjuvant chemotherapy is the modality recommended by both the Asian and American guidelines [24]. Recently, neoadjuvant chemotherapy has been proposed as a promising approach to improve survival compared with the adjuvant modality. Several phase III European studies have demonstrated that the administration of neoadjuvant chemotherapy prior to curative surgery and adjuvant chemotherapy in patients with GC has increased their survival rates [25, 26]. Another theoretical advantage of neoadjuvant chemotherapy is the greater probability that a multimodality approach can be successfully completed, because chemotherapy is given before any possible postoperative complications following extended surgery can develop [27]. In some patients, postoperative adjuvant chemotherapy is restricted owing to surgical complications [28].

There is an increasing interest in the safety and efficacy of LG after neoadjuvant chemotherapy. However, few studies compared the oncological outcomes of LG and OG after neoadjuvant chemotherapy. The edema and fibrotic tissue changes caused by chemotherapy present new technical challenges for laparoscopic treatments [29, 30]. Nevertheless, many investigators have excluded patients receiving chemotherapy from studies of LG for GC. Therefore, we investigated and compared the oncological outcomes of LG and OG after neoadjuvant chemotherapy in patients with locally advanced GC.

## Materials and methods

**Fig. 1:**
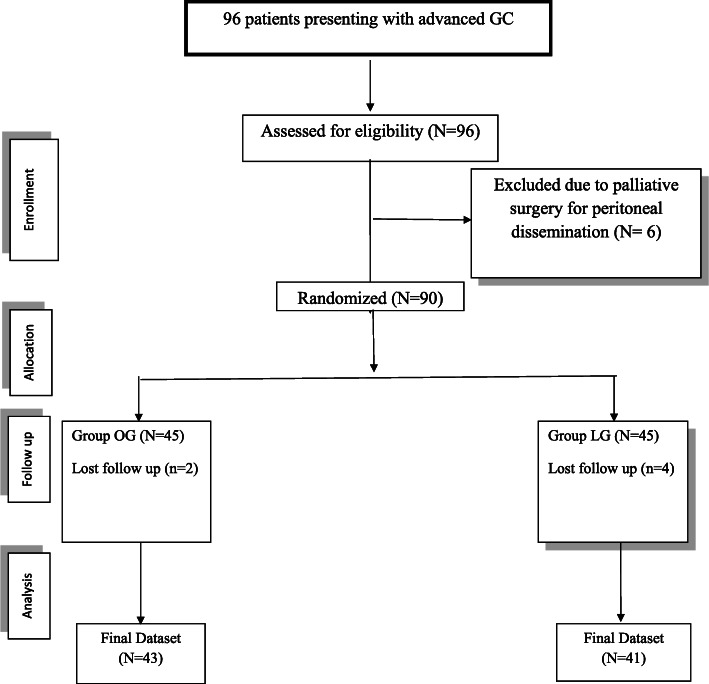
Study flowchart

We conducted a retrospective chart review of all adult patients (≥ 18 years) of both sexes who were diagnosed with locally advanced GC and who underwent either OG or LG at Suez Canal University Hospital, Cairo University Hospitals, and Ramon y Cajal University Hospital. We excluded patients with distant metastasis or other primary malignancies as well as patients who required conversion from laparoscopic to open surgery in order to standardize the variables of the two arms of the study. The study’s protocol received ethical approval from the responsible steering committee. A total of 96 patients who matched our inclusion criteria were initially screened as candidates for this study. Six patients were excluded because they underwent palliative surgery for peritoneal dissemination, so that 90 patients were evaluated in the retrospective review. The final analysis of the included cases is illustrated in Fig. 1; 84 patients (43 OG and 41 LG) were available by the end of the study.

The following were the criteria for patient selection after explanation for all patients the advantages and disadvantages of both techniques:

- The own patient preferences were the solely and main criteria for selection.
- If the patient did not decide which procedure is preferred, a senior operating consultant was one responsible to choose the technique with consideration of 1:1 ratio.

### Preoperative staging

Preoperatively, we took a full patient history and performed a thorough clinical examination of all patients. In addition, we collected the findings of routine laboratory investigations, contrast-enhanced abdominal computed tomography, and upper endoscopy with tissue biopsy. Patients were clinically staged according to the TNM classification, 7th Edition [31]. The neoadjuvant treatment regimen was conducted for T3, T4 tumors, or T2 with associated lymphadenopathy at preoperative staging. The regimen consisted of capecitabine (500 mg/m^2^ orally twice a day) plus oxaliplatin (130 mg/m^2^) for three cycles (21 days in each cycle). This was repeated 6 to 12 weeks after the operation for four cycles. The radiological response to the neoadjuvant chemotherapy was assessed according to the Response Evaluation Criteria in Solid Tumors (version 1.1) [32]. The severity of the chemotherapy-associated adverse events was assessed according to the recommendations of the Common Terminology Criteria for Adverse Events (version 4.0) [33].

### Surgical technique

The surgery was performed within 4 to 6 weeks after the completion of the chemotherapy by a senior expert surgeon in LG. Prophylactic 3rd generation cephalosporin antibiotics were given simultaneously after general anesthesia to all patients, and a Foley catheter was inserted.

A standard LG or OG with appropriate lymphadenectomy according to the Japanese classification of gastric carcinoma (including lymph nodes 1–9, 11p, and 12a in D2 lymphadenectomy and 1–8a and 12a in extended D1 lymphadenectomy) was performed by an experienced surgeon [34].

In both OG and LG groups, an initial exploration was conducted to assess the feasibility of the resection. In the OG group, a 20- to 25-cm midline incision was made from the xiphoid process to the periumbilical area. In the LG group, 4-mm periumbilical ports were inserted in the left upper quadrant and the right and left flank areas. Another 5-mm port was inserted in the right upper quadrant. In both groups, the decision to perform either a subtotal or total resection was based solely on the tumor site and extent. In cases in which the upper one-third of the greater curvature was involved, the spleen was resected. Roux-en-Y procedures, with functional side-to-side anastomoses, were performed to restore the continuity of the gastrointestinal tract. The specimen was pulled out through a small median incision under the xiphoid (approximately 6–8 cm).

Postoperative management was done according to the participating hospitals’ guidelines. Patients were discharged after > 2 days of soft diet without fever or abdominal pain. The adjuvant regimen started on the beginning of the seventh postoperative week and consisted of oxaliplatin plus capecitabine for five cycles. Dose reduction or treatment discontinuation was attempted in cases of serious adverse events. In addition, oxaliplatin was stopped if there were neurological complications. Palliative and supportive care was offered as needed for disease-related symptoms.

### Follow-up and study end points

Patients were followed-up during their hospital stay and for 3 years after the procedure. The primary objective was to compare the 3-year survival rate and overall survival (OS) between the LG and OG groups. Secondary end points included survival time, 3-year recurrence rate, disease progression-free survival (DFS), operative time, intraoperative blood loss, hospital stay, and postoperative complications. The complications were assessed using Clavien-Dindo grades, in which 1 of 5 grades was allocated according to the type of management of the complication [35].

### Statistical analysis

Statistical data analysis was conducted using Microsoft Excel 2013 (Microsoft Corp., Redmond, WA) 32-bit software. Continuous data were expressed as means (± standard deviation [SD]), and categorical data were described as percentages. Comparisons between qualitative data were performed using the chi-square or Fisher’s exact tests, whereas comparisons between quantitative data were performed using the Mann–Whitney or analysis of variance tests. A *P* value of < 5% was considered statistically significant.

## Results

In the current study, the mean age (± SD) of the evaluated patients was 64.0 ± 10.7 years in the OG group and 62.3 ± 4.5 years in the LG group (*P* = 0.45). There was male predominance in the OG group but not in the LG group (60.5% and 48.8%, respectively; *P* = 0.29). Additionally, there were no significant differences between the groups in terms of the tumor site (*P* = 0.28), tumor differentiation (*P* = 0.15), and clinical stage (*P* = 0.52). On the other hand, the frequency of radiological complete response was significantly higher in the OG group than that in the LG group (39.5% vs. 24.4%, respectively; *P* = 0.002) (Table 1).

**Table 1:** Preoperative data of the studied gastrectomy groups

Variables	OG Group (***n*** = 43)	LG Group (***n*** = 41)	***P*** value
Age (mean ± standard deviation), years	64 ± 10.7	62.29 ± 4.5	0.45
Male, no. (%)	26 (60.5%)	20 (48.8%)	0.29
Comorbidity			
• No associated comorbidity	19 (44.2%)	20 (48.7%)	0.19
• Hypertension	9 (21%)	10 (24.5%)	
• Diabetes	8 (18.5%)	8 (19.5%)	
• Bronchial asthma	4 (9.3%)	1 (2.5%)	
• Others	3 (7%)	2 (4.8%)	
Tumor site			
• Esophagogastric junction	6 (14%)	9 (22%)	0.28
• Fundus	2 (4.7%)	4 (9.8%)	
• Body	21 (48.8%)	11 (26.8%)	
• Antrum	12 (27.9%)	13 (31.7%)	
• Pylorus	2 (4.7%)	4 (9.8%)	
Tumor differentiation			
• Well	8 (18.6%)	4 (9.8%)	0.15
• Moderate	10 (23.3%)	15 (36.6%)	
• Poor	22 (51.2%)	22 (53.7%)	
Tumor stage			
• II	15 (34.9%)	15 (36.6%)	0.52
• III	28 (65.1%)	26 (63.4%)	
T stage			
• T2	12 (27.9%)	10 (24.3%)	0.56
• T3	17 (39.5%)	20 (48.7%)	
• T4a	11 (47.2%)	11 (26.8%)	
• T4b	3 (6.9%)	0 (0.0%)	
N stage			
• N0	12 (27.9%)	21 (51.2%)	0.14
• N1	9 (20.9%)	9 (22%)	
• N2	10 (23.3%)	7 (17.1%)	
• N3a	8 (18.6%)	4 (9.8%)	
• N3b	3 (7%)	0 (0.0%)	
Radiological response			
• CR	17 (39.5%)	10 (24.4%)	0.002*
• PD	14 (32.6%)	9 (22.0%)	
• SD	12 (27.9%)	10 (24.4%)	

NOTE: *CR* complete response, *LG* laparoscopic gastrectomy, *OG* open gastrectomy, *PD* progressive disease, *SD* stable disease

*P < 0.05, statistically significant

In terms of intraoperative characteristics, intraoperative blood loss was significantly lower in the LG group than in the OG group (70.5 ± 28.1 mL vs. 157.2 ± 17.6 mL, respectively; *P* = 0.012). No significant differences were detected between the OG and LG groups regarding the operation time (*P* = 0.202), extent of resection (*P* = 0.19), margin of resection (*P* = 0.64), number of total lymph nodes (*P* = 0.17), and number of positive lymph nodes (*P* = 0.14) (Table 2). There were only four patients in whom LG was converted to open surgery because of marked adhesions and difficult anatomical orientation and only one case because of a large matted lymph node in station VIII, which was difficult to dissect.

**Table 2:** Intraoperative data of the studied gastrectomy groups

Variables	OG group (***n*** = 43)	LG group (***n*** = 41)	***P*** value
Duration of operation, min (mean ± SD)	279.9± 70.8	297.8 ± 56.2	0.202
Extent of resection, no. (%)			
• Distal subtotal	16 (37.2%)	15 (36.6%)	0.19
• Total	27 (62.8%)	26 (63.4%)	
Margin of resection, no. (%)			
• R0	40 (93%)	37 (90.2%)	0.64
• R1	3 (7%)	4 (9.8%)	
Type of positive margin, no. (%)			
• Proximal	2 (4.7%)	2 (4.9%)	0.78
• Distal	1 (2.3%)	2 (4.9%)	
Lymphadenectomy type, no. (%)			
• D1+	16 (37.2%)	16 (39%)	0.142
• D2	21 (48.8%)	22 (53.6%)	
• D2+	6 (14%)	3 (7.3%)	
Blood loss, mL (mean ± SD)	157.2 ± 17.65	70.5 ± 28.12	0.012
No. of total lymph nodes (mean ± SD)	27.6 ± 16.5	21.6 ± 10.3	0.17
No. of positive lymph nodes (mean ± SD)	4.4 ± 8	2.9 ± 4.4	0.14

NOTE: *LG* laparoscopic gastrectomy, *OG* open gastrectomy, *SD* standard deviation

The hospital stay was significantly shorter in the LG group than in the OG group (4.75 ± 5.17 days vs. 8.11 ± 2.44 days, respectively; *P* = 0.026). The mortality rate was comparable for patients in the OG group and the patients in the LG group (9.7% vs. 4.6%, respectively; *P* = 0.36). Septic peritonitis and anastomosis leakage were the causes of death in two patients in the OG group, whereas the cause of death was general and not related directly to the operative bed (e.g., myocardial infarction and pulmonary embolism) in the rest of the patients. Patients in the LG group showed a lower rate of postoperative complications; however, this did not reach the level of statistical significance (*P* = 0.16). The types of postoperative complications were comparable between the two groups (*P* = 0.128). Patients in the LG group were less likely to experience high Clavien-Dindo grade complications than patients in the OG group (*P* = 0.026) (Table 3).

**Table 3:** Postoperative data of the studied gastrectomy groups

Variables	OG group (***n*** = 43)	LG group (***n*** = 41)	***P*** value
Hospital stay, days (mean ± SD)	8.11 ± 2.44	4.75 ± 5.17	0.026
Mortality, no. (%)	4 (9.7%)	2 (4.6%)	0.36
Postoperative complications, no. (%)	7 (17.1%)	8 (19.5%)	0.16
Type of surgical complications, no. (%)			
• Abdominal collection	0 (0%)	2 (4.7%)	0.128
• Esophagojejunal leak	4 (9.8%)	3 (7%)	
• Gastrointestinal bleeding	1 (2.4%)	0 (0%)	
• Wound infection	0 (0%)	1 (2.3%)	
• Intraperitoneal bleeding	1 (2.4%)	0 (0%)	
• Pancreatic leak	0 (0%)	2 (4.7%)	
Type of medical complications, no. (%)			
• Urinary tract infection	2 (4.6 %)	0 (0%)	0.227
• Enteritis	1 (2.3%)	0 (0%)	
• Gastrointestinal bleeding	0 (0%)	1 (2.4%)	
• Pleural effusion	(7%)	1 (2.4%)	
• Pulmonary embolism	1 (2.3%)	0 (0%)	
• Sepsis	0 (0%)	1 (2.4%)	
Clavien-Dindo class, no. (%)			
• Grade II	2 (4.9%)	8 (18.6%)	0.026
• Grade IIIA	1 (2.4%)	4 (9.3%)	
• Grade IIIB	2 (4.9%)	0 (0%)	
• Grade IVA	1 (2.4%)	0 (0%)	
• Grade V	2 (4.9%)	0 (0%)	
Reintervention, no. (%)	2 (4.7%)	2 (4.9%)	0.96

NOTE: *LG* laparoscopic gastrectomy, *OG* open gastrectomy, *SD* standard deviation

**Fig. 2:**
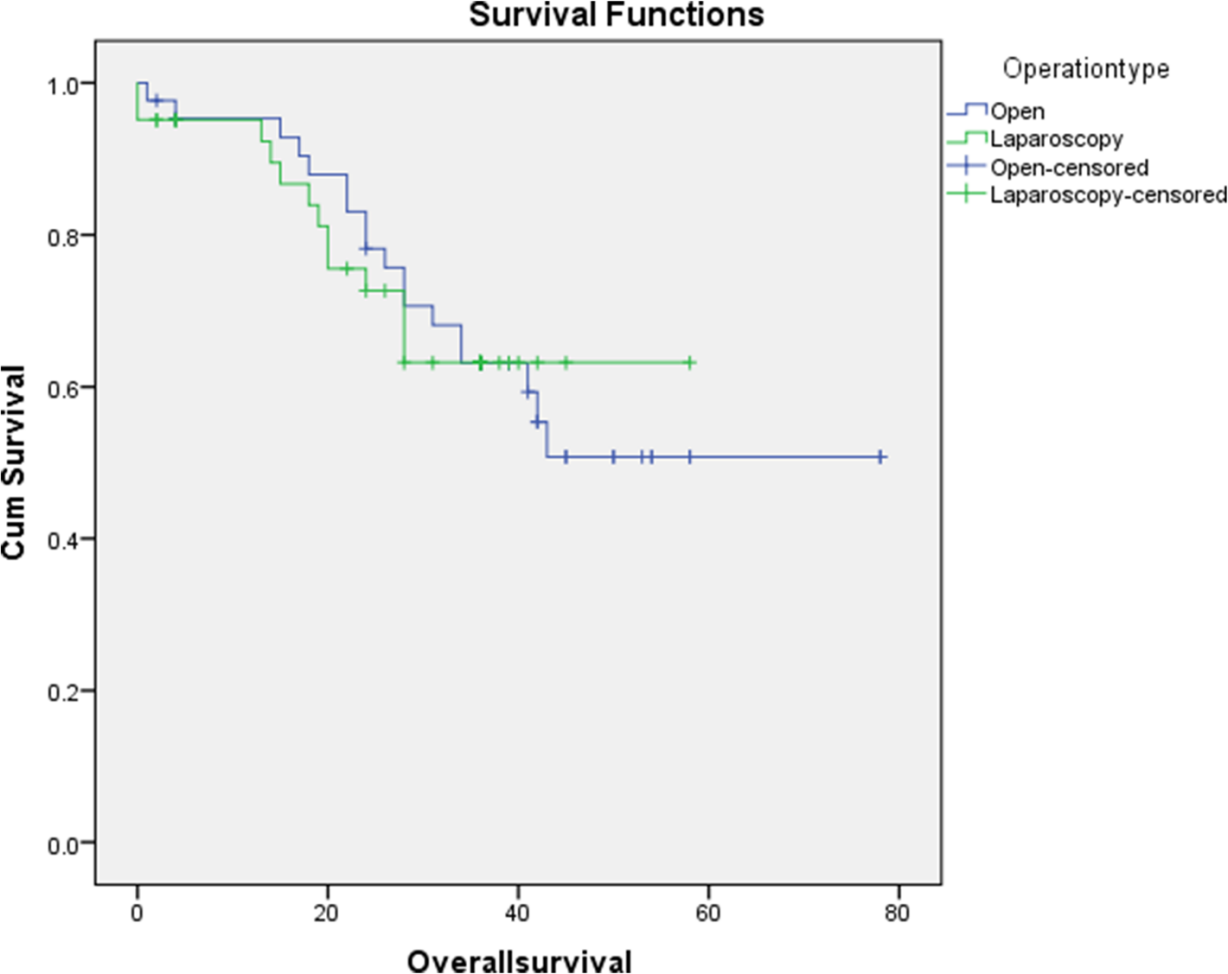
Kaplan–Meier curve showing overall survival

**Fig. 3:**
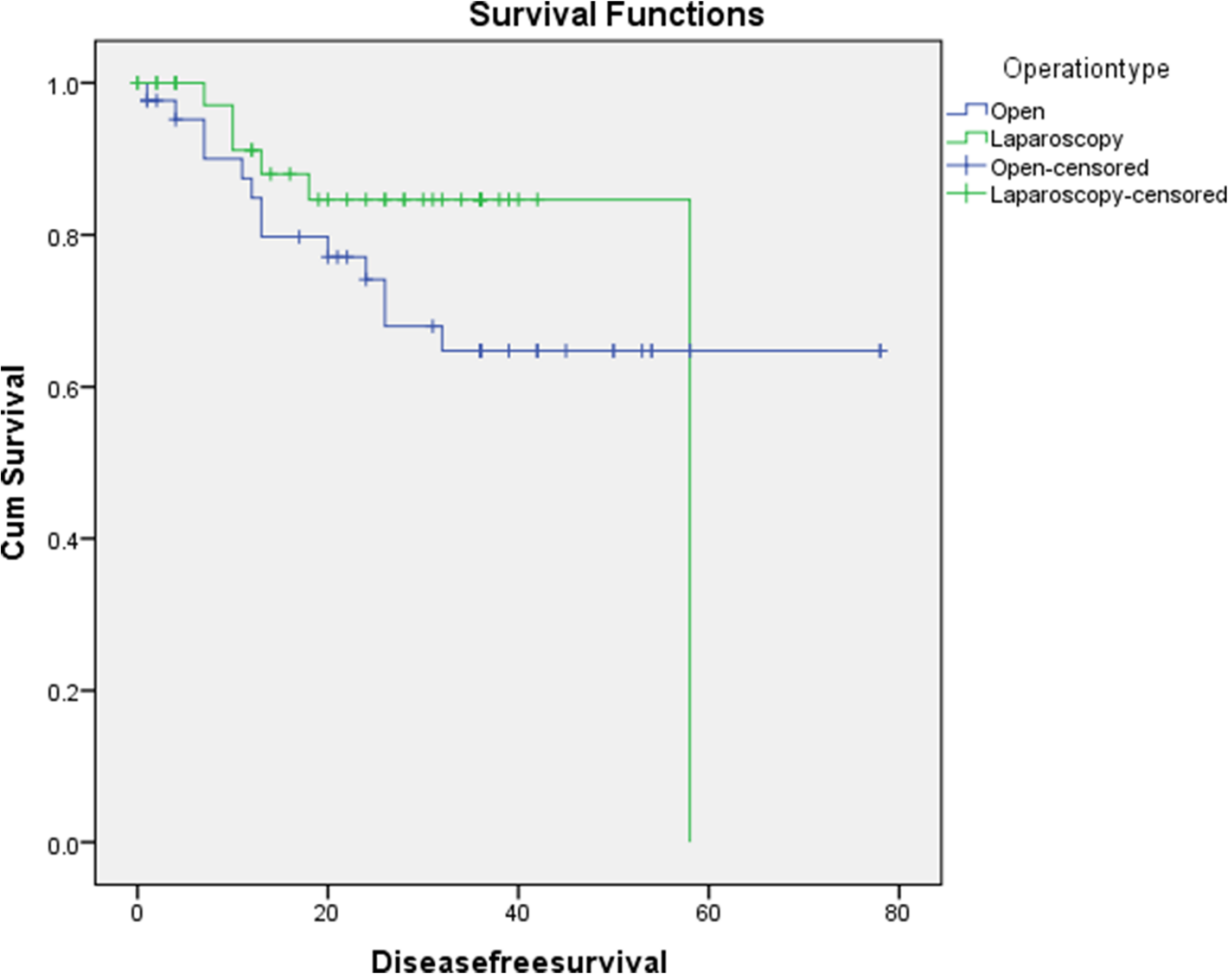
Kaplan–Meier curve showing disease progression-free survival

Regarding long-term outcomes, the 3-year survival rate was comparable between the OG and LG groups (58.1% vs. 68.3%, respectively; *P* = 0.23). Similar trends were observed for the 3-year recurrence rate (*P* = 0.15) and metastasis (*P* = 0.26) (Table 4). The mean survival time was 52.9 months (95% confidence interval [CI], 44.2–61.6) in the OG group vs. 43.3 months (95% CI, 36.6–49.8) in the LG group (*P* = 0.96) (Fig. 2). Likewise, the mean DFS was 56.1 months (95% CI, 46.4–65.8) in the LG group vs. 50.9 months (95% CI, 44.6–57.2) in the OG group (*P* = 0.218) (Fig. 3).

**Table 4:** Three year outcomes of the studied gastrectomy groups

Variables	OG group (***n*** = 43)	LG group (***n*** = 41)	***P*** value
Metastasis, no. (%)			
• Locoregional	8 (18.6%)	2 (4.9%)	0.26
• Liver	1 (2.3%)	1 (2.4%)	
• Carcinomatosis	4 (9.3%)	2 (4.9%)	
• Anastomosis	1 (2.3%)	0 (0.0%)	
Recurrence (no., %)	13 (30.3%)	6 (14.6%)	0.15
Overall survival (no., %)	25 (58.1%)	28 (68.3%)	0.23

NOTE: *LG* laparoscopic gastrectomy, *OG* open gastrectomy, *SD* standard deviation

Cox regression analysis demonstrated that none of the perioperative characteristics was an independent predictor of OS. On the other hand, age younger < 70 years old (hazard ratio, 0.015; 95% CI, 0–0.65) was an independent predictor of favorable DFS (Table 5).

**Table 5:** Cox regression analysis of predictors of overall survival (OS) and disease progression-free survival (DFS)

Variables	OS		DFS	
	HR (95% CI)	***P*** value	HR (95% CI)	***P*** value
Age ≥ 70 years	1.16 (.995–1.25)	0.06	1.152 (1.025–1.29)	0.017
Male sex	0.584 (0.062–5.466)	0.63	0.736 (0.041–13.287)	0.83
Differentiation (moderate)	0.749 (0.007–78.71)	0.93	1.117 (0.001–17.07)	0.39
Differentiation (poor)	1.277 (0.018–4.249)	0.35	1.084 (0.002–2.86)	0.16
Tumor stage III	1.66 (0.23–1.92)	0.45	1.228 (0.34–1.2)	0.081
Radiological response (CR vs. SD/PD)	0.65 (025–1.69)	0.37	0.31 (0.06–2.8)	0.285
Laparoscopic gastrectomy	0.685 (0.112–4.201)	0.68	1.879 (0.092–38.47)	0.113
Total gastrectomy (distal vs. total)	1.299 (0.234–7.198)	0.76	3.9 (0.34–46.45)	0.274
Locoregional metastasis	1.081 (0.1–42.427)	0.43	4.842 (0.075–313.77)	0.45
Distant metastasis	4.53 (0.58–35.35)	0.15	23.479 (0.982–561.12)	0.051
R1 marginal resection	2.45 (0.29–20.29)	0.41	0.019 (0–2.4)	0.111
Complications (yes vs. no)–	0.168 (0.014–1.99)	0.16	2.470 (0.171–35.74)	0.51
Reintervention (yes vs. no)	35.56 (3.39–372.19)	0.003	2.934	0.997
Laparoscopic gastrectomy	1.016 (0.49–2.098)	0.96	0.858 (0.418–1.759)	0.67

NOTE: *CI* confidence interval, *CR* complete response, *HR* hazard ratio, *SD* stable disease, *PD* progressive disease

## Discussion

Owing to the aggressive nature of the disease, old age in the majority of cases, poor nutrition, extreme radical dissection, and surgical trauma, patients with locally advanced GC are prone to prolonged hospital stays, postoperative morbidity, increased financial burden, and even a high risk of postoperative mortality [36]. Thus, in patients with GC, surgeons must take care when choosing the treatment strategy [36]. This makes LG the fastest growing minimally invasive procedure for patients with GC [37]. Several trials have shown that LG is associated with smaller incisions, reduced bleeding, and decreased surgical stress [38, 39]. However, despite the great advances in this technique and its impact on oncological outcomes, LG has some issues, such as decreased intraoperative lung compliance owing to the establishment of artificial pneumoperitoneum as well as the relatively long operative time required for this procedure [40]. Therefore, some researchers have suggested using neoadjuvant chemotherapy before LG or OG because the chemotherapy may help in downstaging the tumor by reducing the tumor size and making R0 resection easier. In addition, micrometastatic tumor cell eradication can begin at an early stage, which is an important advantage over adjuvant chemotherapy [28, 41]. However, little is known about the oncological outcomes of LG and OG after neoadjuvant chemotherapy.

Our study highlighted the differences in the oncological outcomes between LG and OG in two groups of matched patients with GC. In agreement with the literature, our findings showed that LG and OG were comparable in terms of 3-year survival, mean survival time, 3-year recurrence rate, and metastasis. LG had a higher DFS, but this was not statistically significant. The Korean Laparoendoscopic Gastrointestinal Surgery Study trial demonstrated that laparoscopic distal gastrectomy and open distal gastrectomy were almost similar in terms of 5-year survival and 5-year cancer-specific survival rates. Both groups were comparable concerning total deaths and recurrence [38]. There were doubts about the oncological safety of LG for GC, as the risk of locoregional recurrence was potentially increased owing to insufficient lymphadenectomy [14]. An RCT conducted by Hu et al. showed similar rates of D2 lymphadenectomy for LG and OG and comparable postoperative morbidity and mortality [42]. In agreement with our findings, Yu et al. showed a similar 3-year DFS rate for LG and OG in patients with locally advanced GC. Furthermore, the 3-year OS rate, recurrence rate, and mortality rate were comparable for the two groups [22]. In the retrospective analysis by Fujisaki et al., they reported comparable 5-year DFS and OS in the LG and OG groups, respectively [43]. Best et al. found no significant difference in short- and long-term results between LG and OG [44]. The findings of our results and previous trials should be interpreted cautiously. Our study was based on the experience of three surgical centers only and, hence, the generalizability of its findings is limited. Previous reports demonstrated that surgeon’s experience and preparedness of the healthcare facility play a significant role in the outcomes of LG [45]. Thus, future studies with multicenter collaboration are needed to control for the influence of surgical experience. Besides, future studies should also assess the value of robotic surgery in the era of neoadjuvant chemotherapy for patients with GC [46].

Notably, Li et al. showed that after four cycles of neoadjuvant chemotherapy, LG and OG were comparable in terms of distal and proximal margins, number of resected or metastatic lymph nodes, postoperative complications, operative time, blood loss, and length of hospital stay [28]. After 3 years, they published an RCT showing that, among 95 patients with GC who were receiving neoadjuvant chemotherapy before surgery, the LG group had a substantially lower postoperative complication rate than that of the OG group. Moreover, LG was associated with a lower postoperative pain score (visual analog scale) compared with that of OG [47]. Wu et al. compared two groups of GC patients. The first group received neoadjuvant chemotherapy before undergoing surgery, and the second group was assigned to surgery directly. Total blood loss in the neoadjuvant group was substantially higher than that of the other group. However, operative time, lymph nodes harvested, multiorgan resection, and postoperative complications were comparable between the two groups [48]..

Concerning the intra and postoperative outcomes, we found that the LG was associated with less intraoperative blood loss, shorter hospitalization, and a lower rate of postoperative complications. On the other hand, the in-hospital mortality rate and types of postoperative complications were comparable in both groups. Recent meta-analyses showed that LG was associated with decreased intraoperative blood loss, shorter postoperative hospital stay and shorter time to first oral intake compared with results for OG. On the other hand, LG had a longer operative time and comparable postoperative mortality rate compared with OG [12, 49].

Anastomotic leakage and septic peritonitis are considered the major complications of gastric surgery. In our study, these two complications were the causes of death of two patients in the LG group. Hu et al. reported the anastomotic leakage rate in their LG group was 1.9% [42], which was within the previously reported range [21, 23, 50, 51]. This differed from the research results of Rod et al., who reported a high anastomotic leakage rate in the LG group (17%), especially in comparison with the rate in the OG group (10%). The overall incidences of postoperative complications and surgical complications were higher in the LG group than in the OG group, but postoperative mortality did not differ significantly between the groups [52]. Similarly, Haverkamp et al. reported a 37% complication rate in their LG group [53].

The present study gives novel insights about the oncological outcomes of LG and OG after neoadjuvant chemotherapy in patients with locally advanced GC. Nonetheless, we acknowledge that the present study has several limitations. The study was retrospective in nature; hence, our study was prone to misclassification and ascertainment bias. Besides, patients’ records were collected by convenience sampling technique, which might have increased the risk of selection bias. The baseline characteristics of LG and OG groups were not comparable in terms of radiological response regarding equally distributed comorbidities and expert surgeons in both LG and open group, which did not affect the postoperative outcomes of the patients. Finally, the study was based on the experience of few surgical centers only, which may affect the generalizability of our findings. However, it adds some value due to multicentricity of the study in 2 different countries.

## Conclusion

LG for patients with locally advanced GC who have received neoadjuvant chemotherapy is a safe and feasible alternative to OG. LG showed reduced blood loss, better postoperative healing, and lower postoperative morbidity relative to OG. However, the oncological outcomes remained comparable between both groups. These findings indicated that LG had more favorable intra- and postoperative outcomes in terms of safety and tolerability. However, the efficacy of LG compared with OG remains controversial. The direct impact of neoadjuvant chemotherapy on LG or OG should be investigated by comparing patients who received neoadjuvant therapy before surgery with those who were assigned to surgery directly. Owing to the limitation of the present study, future well-controlled trials with multinational collaboration are needed.

## Supplementary Information

**Additional file 1: Supplementary No.1.** Coefficients from univariable models of OS.

## Data Availability

The datasets used and/or analyzed during the current study are available from the corresponding author on reasonable request. All data generated or analyzed during this study are included in this published article [and its supplementary information files].

## Abbreviations

CI: Confidence interval
GC: Gastric cancer
LG: Laparoscopic gastrectomy
MD: Mean difference
OG: Open gastrectomy
RCT: Randomized controlled trial
SD: Standard deviation
