# Supplementary material for: Oncological outcomes of laparoscopic versus open gastrectomy after neoadjuvant chemotherapy for locally advanced gastric cancer: a retrospective multicenter study
Source: World J Surg Oncol. 2021 Jul 9;19:206. doi: 10.1186/s12957-021-02322-2 (PMC8272390; doi:10.1186/s12957-021-02322-2)
Supplement: Supplementary file 1 — Additional file 1: Supplementary No.1. Coefficients from univariable models of OS. [file 12957_2021_2322_MOESM1_ESM.docx]

**Supplementary No.1** Coefficients from univariable models of OS

| **Variables** | **OS** | |
| --- | --- | --- |
|  | **HR (95% CI)** | ***P* value** |
| Age ≥70 y | 1.028 (0.993 -1.06) | 0.119 |
| Male gender | 2.3 (0.062 - 5.466) | 0.93 |
| Differentiation (moderate) | 1.084 (0.36 - 3.25) | 0.86 |
| Differentiation (poor) | 0.913 (0.331 -2.51) | 0.88 |
| Tumor stage III | 2.36 (0.96 -5.77) | 0.95 |
| Radiological response (CR vs SD/PD) | 0.65 (025–1.69) | 0.37 |
| Laparoscopic Gastrectomy | 1.016 (0.49 - 2.09) | 0.96 |
| Total gastrectomy (distal vs total) | 2.89 (0.635 - 13.16) | 0.17 |
| Loco-regional Metastasis | 1.61 (0.33 - 7.77) | 0.54 |
| Distant Metastasis | 1.41 (0.45 - 4.33) | 0.55 |
| R1 Marginal resection | 2.45 (0.29–20.29) | 0.41 |
| Complications (yes vs no) | 0.168 (0.014 - 1.99) | 0.16 |
| Reintervention (yes vs no) | 35.56 (3.39–372.19) | 0.003 |
